# Targeting complement anaphylatoxin C5a receptor in hyperoxic lung injury in mice

**DOI:** 10.3892/mmr.2014.2394

**Published:** 2014-07-18

**Authors:** YING XU, ZHE TIAN, PEIYU XIE

**Affiliations:** 1 Respiratory Department of Heilongjiang Provincial Hospital, Harbin, Heilongjiang 150001, P.R. China; 2 Department of Molecular Genetics, Graduate School of Medical Sciences, Kumamoto University, Kumamoto 860-8556, Japan; 3 Department of Molecular Physiology, Faculty of Life Sciences, Kumamoto University, Kumamoto 860-8556, Japan

**Keywords:** hyperoxic lung injury, complement anaphylatoxin C5a, receptor antagonist, macrophage

## Abstract

Receptor binding of complement anaphylatoxin C5a results in proinflammatory activation of numerous diseases, but the role of receptor-mediated action during hyperoxic lung injury has, to the best of our knowledge, not yet been investigated. The contribution of the C5a receptor (C5aR) to hyperoxic lung injury in mice was investigated in this study. The effect of C5aR on hyperoxic lung injury in Balb/c mice was examined employing a C5aR antagonist (C5aRA). The mice were ventilated with 100% oxygen for 36 h with or without the administration of C5aRA. C5aR expression levels in non-treated or 100% oxygen-treated mice were assessed by reverse transcription polymerase chain reaction (RT-PCR) and flow cytometry. The body weight and the relative lung weight of the mice, and the morphological changes in the lung were then determined. The total cell counts and the number of macrophages, neutrophils and lymphocytes in bronchoalveolar lavage fluid (BALF) were determined using cytocentrifuge slides and a hemocytometer. The levels of interleukin-6 (IL-6), monocyte chemotactic protein (MCP-1) and tumor necrosis factor-α (TNF-α) in BALF and the myeloperoxidase (MPO) activity in the lung tissue were measured by enzyme-linked immunosorbent assay. The relative levels of CD68 and F4/80 messenger ribonucleic acid (mRNA) expression in the lung tissue were detected by RT-PCR. The TNF-α, IL-6 and MCP-1 protein expression levels in the lung tissue were assessed by western blot analysis. The results revealed hyperoxia-induced morphological changes, lung injury and increased expression levels of C5aR in lung tissue. The hyperoxia-induced increases in the total cell count and the number of macrophages, neutrophils and lymphocytes in the BALF were all significantly reduced in the mice receiving C5aRA. Treatment with C5aRA also attenuated the morphological changes and reduced MPO activity, and CD68 and F4/80 mRNA expression levels in the lung tissue, as well as the levels of IL-6, MCP-1 and TNF-α in BALF and lung tissue. In conclusion, C5a-C5aR action accelerated hyperoxia-induced lung injury, but this hyperoxic lung injury was attenuated by treatment with C5aRA.

## Introduction

Interest in complement molecules has intensified in a number of diseases (1). In particular, complement anaphylatoxin C5a, an anaphylatoxin liberated from the N-terminal region of the parental protein α-chain, has been investigated (2). C5a is a potent soluble anaphylotoxic and chemotactic inflammatory regulator, inducing the recruitment and activation of neutrophils and monocytes/macrophages. C5a specifically binds to the C5a receptor (C5aR) and causes proinflammatory activation (3). In addition to immune cells (neutrophils, monocytes/macrophages, mast cells and T cells), C5aR has also been detected in non-immune cells and in different tissues, such as the lung (4).

High oxygen mechanical ventilation is widely used in clinical therapy, as hypoxemia occurs in various diseases (5). For example, high oxygen mechanical ventilation is an important method for treating serious respiratory failure conditions, such as acute respiratory distress syndrome (ARDS). However, exposure to high levels of oxygen for prolonged periods may result in an inflammatory reaction and lung injury (6). Therefore, a therapeutic strategy alleviating hyperoxia-induced lung injury is important and necessary. Since C5a is a risk factor for lung injury, the role of C5aR-mediated action during hyperoxic lung injury was determined in the present study, along with whether a C5aR antagonist (C5aRA) attenuates this hyperoxia-induced lung injury, through inhibition of the C5aR-mediated action. C5a bound to C5aR on leukocytes and cells of the lung tissue, results in macrophage chemotaxis and provokes the secretion of cytokines and chemokines, such as tumor necrosis factor-α (TNF-α), interleukin-6 (IL-6) and monocyte chemotactic protein (MCP-1) (7), which feedback to macrophages and increase the expression levels of C5aR, further aggravating lung injury (3,8). To the best of our knowledge, the role of C5aR-mediated action during hyperoxic lung injury was analyzed for the first time in the present study.

## Materials and methods

### Animals

The study was approved by the Ethics Committee of the Faculty of Life Sciences, Kumamoto University (Kumamoto, Japan). The animal care and protocol for this study were in accordance with the Animal Experiment Guidelines of Kumamoto University. Balb/c mice (Kyudo Co., Ltd., Saga, Japan), aged six to eight-weeks, were fed normal chew and water *ad libitum*. The mice were ventilated with 100% oxygen for 36 h, as reported previously (9). C5aRA JPE-1375 (1 μg/h/25 g body weight; Jerini AG, Berlin, Germany) was administered via an ALZET mini-osmotic pump (American Health & Medical Supply International Corp., Scarsdale, NY, USA) immediately following the initiation of exposure to 100% oxygen, according to a previously described method (10). Mice were sacrificed 24 h after 100% oxygen exposure and the lung tissue samples were collected. The body weight and the relative lung weight of the mice were determined.

### Reverse transcription polymerase chain reaction (RT-PCR)

Total RNA was extracted from the lung tissue and the relative expression levels of C5aR, CD68 and F4/80 messenger ribonucleic acids (mRNAs) were normalized to those of 18 s. For RT-PCT, the following primers were used: C5aR forward: 5′-GACCCCATAGATAACAGCA-3′ and reverse: 5′-CAGAGGCAACACAAAACCCA-3′; F4/80 forward: 5′-GAGATTGTGGAAGCATCCGAGAC-3′ and reverse: 5′-GATGACTGTACCCACATGGCTGA-3′; CD68 forward: 5′-CATCAGAGCCCGAGTACAGTCTACC-3′ and reverse: 5′-AATTCTGCGCCATGAATGTCC-3′; 18 s forward: 5′-GTAACCCGTTGAACCCCATT-3′ and reverse: 5′-CCATCCAATCGGTAGTAGCG-3′; GAPDH forward: 5′-TTGCCATCAATGACCCCTTCA-3′ and reverse: 5′-CGCCCCACTTGATTTTGGA-3′. RT-PCR was performed using the Applied Biosystems 7300 Fast Real-Time PCR system (Applied Biosystems, Grand Island, NY, USA).

### Preparation of bronchoalveolar lavage fluid (BALF)

Whole lung lavage was performed four times with injections of 0.5 ml sterile saline through a 21G flat syringe needle cannulated 0.7 cm into the trachea. BALF was recovered from each mouse examined and used for quantitative cell counting. A 100 ml aliquot of BALF was used for the total cell count and the remainder was immediately centrifuged at 1,000 g for 10 min. The total quantity of cells was counted using a hemocytometer and cell differentiation was determined for >500 cells placed on cytocentrifuge slides and treated with Wright-Giemsa staining, according to a previously described method (11). Macrophages were isolated from the BALF and C5aR expression levels in BALF macrophages from non-treated and 100% oxygen-treated mice was assessed by flow cytometry. The BALF supernatants were stored at −80°C for cytokine and chemokine analysis.

### Flow cytometry

The macrophages (2×10^6^ cells/ml) obtained from BALF from non-treated or 100% oxygen-treated mice were stained with C5aR antibodies (anti-mouse CD88-phycoerythrin rat IgG2a; Serotec, Oxford, UK) and anti-human CD88-fluorescein isothiocyanate mouse IgG2a (Genway Biotech. Inc., San Diego, CA, USA) antibodies as an isotype control for 30 min on ice. The cells were analyzed in a FACS Calibur flow cytometer (BD Biosciences, Tokyo, Japan).

### Enzyme-linked immunosorbent assay (ELISA)

BALF was collected for the TNF-α, IL-6 and MCP-1 assays, which were conducted using respective mouse ELISA kits (Sigma-Aldrich, Tokyo, Japan). The ELISA plates were coated with 100 μl capture antibody per well at 4°C overnight. Following appropriate washing, 200 μl assay dilution buffer was added per well for blocking at room temperature for 1 h. The sample and serial dilutions of standards were added to the plate and incubated at 4°C overnight. Subsequent to coating with detection antibody, avidin-horse radish peroxidase (HRP) was added and the samples were incubated at room temperature for 30 min. The substrate 3,3′,5,5′-tetramethylbenzidine (TMB) was added and the solution was incubated for 15 min. Subsequently, 2NH_2_SO_4_ was added to stop the reaction and absorbance at 450 nm was measured using an ELISA reader.

### Morphometric analysis

Conventional light microscopic examination of the lung tissue was performed using paraffin-embedded samples: 5 mm sections were stained with hematoxylin and eosin, and assessed in a blinded manner. The microscopical images were captured using an automatic microscope, Provis AX-70 with a camera (Olympus Optical, Tokyo, Japan). Morphometric analyses of the lung samples were performed with NIH 1200 image analysis software as previously described (12).

### Measurement of myeloperoxidase (MPO) activity in lung tissue

MPO activity was measured using a murine MPO ELISA kit (Sigma-Aldrich). Briefly, the pulmonary tissue was homogenized using a tissue rotator in lysis buffer containing 200 mm NaCl, 5 mm EDTA, 10 mm Tris, 10% glycerin, 1 mm phenylmethylsulfonyl fluoride, 1 μg/ml leupeptin and 28 μg/ml aprotinin (pH 7.4), and was centrifuged at 1,700 g for 30 min. The supernatants were incubated in microtiter wells coated with antibodies recognizing mouse MPO. Biotinylated tracer antibody was bound to captured mouse MPO. The streptavidin peroxidase conjugate was added to bind to the biotinylated tracer antibody and react with TMB. The enzyme reaction was stopped by the addition of oxalic acid. The spectrophotometric shift at 460 nm was determined in duplicate using an ELISA reader (MTP-800 Microplate reader; Corona Electric, Tokyo, Japan). The mouse MPO concentrations of the samples were determined from a standard curve.

### Western blotting analysis

Electrophoresis was performed using a vertical slab gel with 12% polyacrylamide content according to a method described by Laemmli (13). The transfer of proteins from SDS-PAGE to a supported nitrocellulose membrane (Bio-Rad, Tokyo, Japan) was performed electrophoretically according to a procedure reported by Kyhse-Andersen (14), with certain modifications, using a Semi-Dry Electroblotter (Sartorious AG, Göttingen, Germany) for 90 min at 15 V electric current. The membrane was treated with Block Ace™ (4%) for 30 min at 22°C. The first reaction was performed using rabbit IgG antibodies against TNF-α, IL-6 and MCP-1 in phosphate-buffered saline containing 0.03% Tween 20 for 1 h at 22°C. Subsequent to washing in the same buffer, the second reaction was performed using HRP-conjugated anti-rabbit IgG goat IgG (20 ng/ml) for 30 min at 22°C. Following washing, an enhanced chemiluminescence (ECL) reaction was performed on the membrane using an ECL Plus Western Blotting Detection system™ (GE Healthcare Life Sciences, Tokyo, Japan).

### Statistical analysis

Data are expressed as the mean ± SD. Each experiment was repeated at least three times. Student’s t-test was used and P<0.05 was considered to indicate a statistically significant difference.

## Results

### Hyperoxia increases C5aR expression levels in the lung

**Figure 1:**
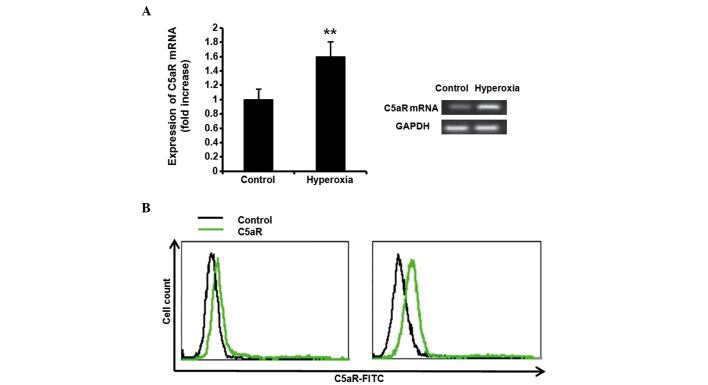
Effect of hyperoxia on C5aR expression levels in the mouse lung. To investigate the effect of hyperoxia on the expression levels of C5aR, Balb/c mice were ventilated with 100% oxygen for 36 h. (A) Following treatment with 100% oxygen, the C5aR mRNA expression levels in the mouse tissue were assessed by reverse transcription polymerase chain reaction. Hyperoxia significantly increased the expression levels of C5aR in the lung tissue. (B) C5aR expression levels in BALF macrophages from non-treated or 100% oxygen-treated mice were also assessed by flow cytometry. The C5aR expression levels in BALF macrophages from 100% oxygen-treated mice were significantly increased. Data are expressed as the mean ± SD (n=5). ^**^P<0.01, hyperoxia vs. control. C5aR, C5a receptor; BALF, bronchoalveolar lavage fluid; FITC, fluorescein isothiocyanate.

To investigate the effect of hyperoxia on the expression levels of C5aR, the Balb/c mice were ventilated with 100% oxygen for 36 h. Following treatment with 100% oxygen, the C5aR mRNA expression levels in the mice tissue was assessed by RT-PCR. Hyperoxia significantly increased the expression levels of C5aR in the lung tissue (Fig. 1A, P<0.01). The C5aR expression levels in BALF macrophages from non-treated or 100% oxygen-treated mice were also assessed by flow cytometry and the C5aR expression levels in the 100% oxygen-treated mice were significantly increased, compared with those in the non-treated mice (Fig. 1B).

### C5aRA reduces hyperoxia-induced body weight and lung weight changes

**Figure 2:**
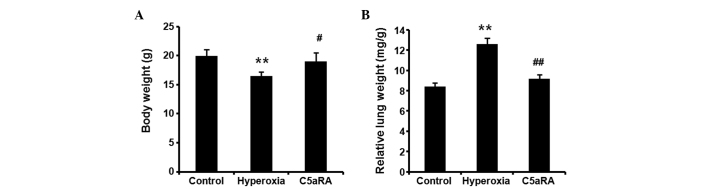
C5aRA reduced the hyperoxia-induced body weight and lung weight changes. Following treatment with 100% oxygen for 36 h with or without C5aRA treatment, (A) the body weights of the mice were significantly reduced, and (B) the relative lung weights were significantly increased. (A and B) Hyperoxia-induced body weight and relative lung weight changes were significantly reduced in C5aRA treatment mice. Data are expressed as the mean ± SD (n=5). ^**^P<0.01, hyperoxia vs. control; ^##^P<0.01, ^#^P<0.05, hyperoxia + C5aRA vs. hyperoxia. C5aRA, C5a receptor antagonist.

The Balb/c mice were ventilated with 100% oxygen for 36 h with or without C5aRA treatment. The body weights of the mice were significantly reduced (Fig. 2A, P<0.01) and the relative lung weight was significantly increased (Fig. 2B, P<0.01) following hyperoxia alone. However, the hyperoxia-induced body weight and relative lung weight changes were significantly reduced in the C5aRA-treated mice (Fig. 2A and B).

### C5aRA attenuates the hyperoxia-induced inflammatory reaction in BALF

**Figure 3:**
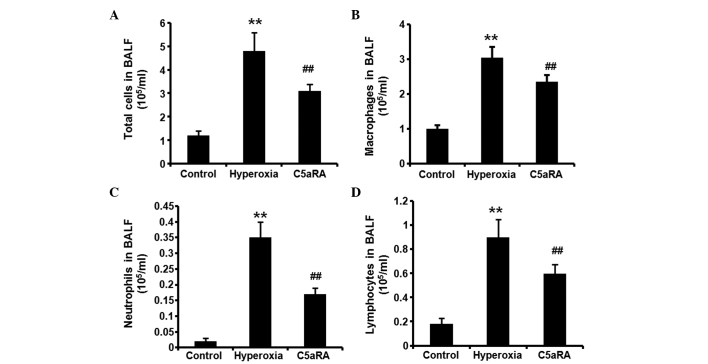
C5aRA attenuated hyperoxia-induced total cell counts and the number of macrophages, neutrophils and lymphocytes in BALF. Following treatment with 100% oxygen for 36 h with or without C5aRA treatment, BALF was collected. The total cell count and the number of macrophages, neutrophils and lymphocytes in BALF were determined by examination of cytocentrifuge slides and hemocytometer. (A) Hyperoxia increased the total cell count and the number of (B) macrophages, (C) neutrophils and (D) lymphocytes in BALF from 100% oxygen-treated mice. The hyperoxia-induced increases in total cell counts and the number of macrophages, neutrophils and lymphocytes in BALF were all significantly reduced in mice receiving C5aRA. Data were normalized with cell numbers and are expressed as mean ± SD (n=5). ^**^P<0.01, hyperoxia vs. control; ^##^P<0.01, ^#^P<0.05, hyperoxia + C5aRA vs. hyperoxia. C5aRA, C5a receptor antagonist; BALF, bronchoalveolar lavage fluid.

**Figure 4:**
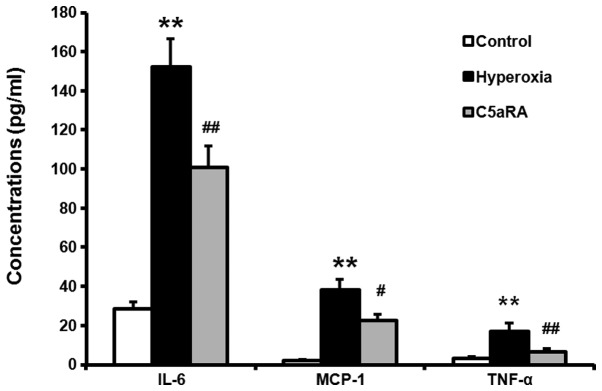
C5aRA attenuated hyperoxia-induced expression of IL-6, MCP-1 and TNF-α in BALF. Balb/c mice were ventilated with 100% oxygen for 36 h with or without C5aRA treatment, and the levels of IL-6, MCP-1 and TNF-α in BALF were measured by ELISA. Hyperoxia significantly induced TNF-α, IL-6 and MCP-1 expression in BALF, and the hyperoxia-induced increases in TNF-α, IL-6 and MCP-1 expression levels were significantly suppressed by the treatment with C5aRA. Data were normalized with cell numbers and are expressed as mean ± SD (n=5). ^**^P<0.01, hyperoxia vs. control; ^##^P<0.01, hyperoxia + C5aRA vs. hyperoxia. C5aRA, C5a receptor antagonist; IL-6, interleukin-6; MCP-1, monocyte chemotactic protein; TNF-α, tumor necrosis factor-α; BALF, bronchoalveolar lavage fluid; ELISA, enzyme-linked immunosorbent assay.

Following treatment with 100% oxygen for 36 h with or without C5aRA treatment, BALF was collected. The total cell counts and the number of macrophages, neutrophils and lymphocytes in BALF were determined by cytocentrifuge slides and hemocytometer. Hyperoxia significantly increased the total cell count (Fig. 3A) and the number of macrophages (Fig. 3B), neutrophils (Fig. 3C) and lymphocytes (Fig. 3D) in BALF from 100% oxygen treated mice, compared with control mice (P<0.01). However, the hyperoxia-induced increases in the total cell count and the number of macrophages, neutrophils and lymphocytes in BALF were all significantly reduced in mice receiving C5aRA (P<0.01). The levels of IL-6, MCP-1 and TNF-α in BALF were measured by ELISA. As shown in Fig. 4, hyperoxia significantly induced TNF-α, IL-6 and MCP-1 expression in BALF (P<0.01), and the hyperoxia-induced TNF-α, IL-6 and MCP-1 expression was significantly suppressed by the C5aRA treatment (P<0.05).

### C5aRA attenuates the hyperoxia-induced inflammatory reaction in lung tissue

**Figure 5:**
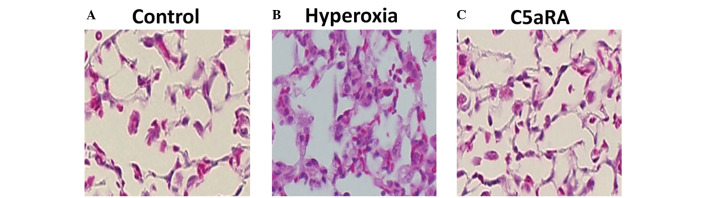
C5aRA attenuated hyperoxia-induced lung morphological changes. Balb/c mice were ventilated with 100% oxygen for 36 h with or without C5aRA treatment, and lung morphological changes were analyzed. (A) Control lung tissue. (B) Hyperoxia-induced lung injury and leukocyte infiltration. (C) C5aRA significantly attenuated lung injury and reduced leukocyte infiltration. Morphometric analyses of lung samples were performed with NIH 1200 image analysis software (n=3). Magnification, ×400 staining, hematoxylin and eosin. C5aRA, C5a receptor antagonist.

Balb/c mice were ventilated with 100% oxygen for 36 h with or without C5aRA treatment and lung morphological changes were analyzed. As shown in Fig. 5, hyperoxia induced lung injury and leukocyte infiltration, but C5aRA treatment significantly attenuated this lung injury and reduced the leukocyte infiltration (P<0.01).

**Figure 6:**
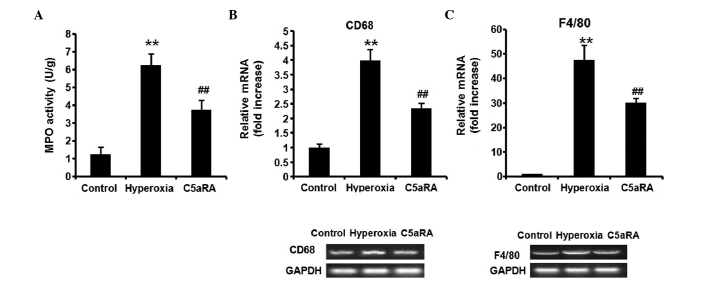
C5aRA attenuated hyperoxia-induced MPO activity, and CD68 and F4/80 expression in the lung tissue. Balb/c mice were ventilated with 100% oxygen for 36 h with or without C5aRA treatment, (A) MPO activity was measured by enzyme-linked immunosorbent assay, and the relative levels of (B) CD68 and (C) F4/80 mRNA expression were detected by reverse transcription polymerase chain reaction. Hyperoxia induced MPO activity, and CD68 and F4/80 mRNA expression in the lung tissue; the hyperoxia-induced MPO activity, and CD68 and F4/80 mRNA expression were significantly suppressed by the treatment with C5aRA. Data are expressed as mean ± SD (n=5). ^**^P<0.01, hyperoxia vs. control; ^##^P<0.01, hyperoxia + C5aRA vs. hyperoxia. C5aRA, C5a receptor antagonist; MPO, myeloperoxidase; mRNA, messenger ribonucleic acid.

**Figure 7:**
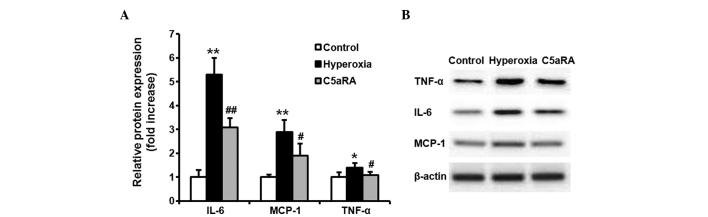
Increased expression levels of TNF-α, IL-6 and MCP-1 in lung tissue were attenuated by C5aRA. Balb/c mice were ventilated with 100% oxygen for 36 h with or without C5aRA treatment. The TNF-α, IL-6 and MCP-1 protein expression levels in the lung tissue were assessed by western blot analysis. The IL-6, MCP-1 and TNF-α protein levels were significantly increased following treatment with 100% oxygen. Treatment with C5aRA significantly reduced IL-6, MCP-1 and TNF-α protein expression levels in the lung tissue (A is a quantification of B). Data are expressed as the mean ± SD (n=5). ^**^P<0.01, ^*^P<0.05, hyperoxia vs. control; ^##^P<0.01, ^#^P<0.05, hyperoxia + C5aRA vs. hyperoxia. TNF-α, tumor necrosis factor-α; IL-6, interleukin-6; MCP-1, monocyte chemotactic protein-1; C5aRA, C5a receptor antagonist.

The MPO activity in the lung tissue was measured by ELISA and the relative levels of CD68 and F4/80 mRNA expression in the lung tissue were detected by RT-PCR. Hyperoxia significantly induced MPO activity (Fig. 6A), and CD68 (Fig. 6B) and F4/80 (Fig. 6C) mRNA expression in the lung tissue (P<0.01). The hyperoxia-induced MPO activity, and the CD68 and F4/80 mRNA expression levels were significantly suppressed by C5aRA treatment (P<0.01). In addition, the TNF-α, IL-6 and MCP-1 protein expression levels in the lung tissue were assessed by western blot analysis and were significantly increased by the treatment with 100% oxygen (Fig. 7, P<0.01). Treatment with C5aRA significantly reduced IL-6, MCP-1 and TNF-α protein levels in the lung tissue, compared with those of the hyperoxia-only group.

## Discussion

In the present study, to the best of our knowledge, for the first time, the role of C5aR-mediated action during hyperoxic lung injury of mice is demonstrated. C5a is an anaphylatoxin liberated from the N-terminal region of the parental protein α-chain and is similar in molecular structure (2). C5a is a potent soluble anaphylotoxic and chemotactic inflammatory mediator promoting the recruitment and activation of neutrophils and monocytes/macrophages (15). C5a acts on numerous types of cells by binding to the C5aR receptor and causes proinflammatory activation (3,16). In addition to immune cells (neutrophils, monocytes/macrophages, mast cells and T cells), C5aR has also been detected in nonimmune cells and in different tissues, including the lung (4). The function of C5a has been investigated in a number of lung diseases. Inhibition of the C5a reaction has been shown to alleviate paraquat-induced acute lung injury (17); however, the acute lung injury induced by lipopolysaccharide is independent of C5a activation (18). Nevertheless, the role of C5a-C5aR-mediated action during hyperoxic lung injury has, to the best of our knowledge not yet been analyzed.

In recent years, high oxygen mechanical ventilation has been widely used in the treatment of the hypoxemia associated with various diseases (5,19). For instance, high oxygen mechanical ventilation is an important method for treating serious respiratory failure conditions, such as ARDS. However, exposure to high levels of oxygen for prolonged periods may result in an inflammatory reaction and lung injury (6). Therefore, a therapeutic strategy alleviating hyperoxia-induced lung injury is required. Since C5a is a risk factor and exerts a critical role in lung injury, the role of C5aR-mediated action in mice during hyperoxic lung injury was determined, along with whether C5aRA inhibits the C5aR-mediated action.

In the present study, the role of C5aR-mediated action during hyperoxic lung injury was analyzed, to the best of our knowledge, for the first time. Hyperoxia was shown to induce lung injury and C5aRA treatment was demonstrated to attenuate this hyperoxia-induced lung injury. Following treatment with 100% oxygen, the expression levels of C5aR were significantly increased in the lung tissue and in BALF macrophages (Fig. 1A and B). Hyperoxia significantly increased the total cell count and the number of macrophages, neutrophils and lymphocytes in BALF, and the increased total cell counts were all significantly reduced in themice receiving C5aRA (Fig. 3). Furthermore, treatment with C5aRA attenuated the hyperoxia-induced morphological changes in the lung tissue (Fig. 5).

Subsequent to treatment with 100% oxygen, inflammatory cells, such as neutrophils, monocytes and macrophages, are recruited into lung tissue (20). In concurrence with this, the data from the present study revealed that hyperoxia induced MPO activity. MPO is a marker of lung neutrophil infiltration and the accumulation of neutrophils is determined by lung MPO content (18) (Fig. 6A). In the present study, CD68 and F4/80 mRNA expression levels in the lung tissue, indicators of macrophage accumulation, were significantly increased following treatment with 100% oxygen (Fig. 6B and C). Treatment with C5aRA significantly reduced MPO activity, and CD68 and F4/80 mRNA expression levels in the lung tissue, compared with the hyperoxia-only group. C5aRA also attenuated the morphological changes induced by hyperoxia (Fig. 4).

C5a binds to receptors on inflammatory cells in the lung, particularly those on the surfaces of macrophages and induces the secretion of cytokines and chemokines, such as TNF-α, IL-6 and MCP-1 (7). TNF-α is a canonical inflammatory cytokine that promotes the inflammatory response (21), and IL-6 is a pleiotropic cytokine involved in pro- and anti-inflammatory responses through the regulation of leukocyte function and apoptosis (22). IL-6 has been reported to beneficially regulate neutrophil adhesion and migration (23); elevated IL-6 levels have been demonstrated in the majority of lung injury models and IL-6 may be a biological marker of lung injury (24,25). MCP-1 is a potent chemoattractant that is essential in various inflammatory diseases involving monocyte/macrophages recruitment (26). These factors may feed back to macrophages and increase the expression levels of C5aR, thus further aggravating lung injury (3,8). In the present study, to improve understanding of the protective mechanism of C5aRA on hyperoxia-induced lung injury, the levels of TNF-α, IL-6 and MCP-1 were examined in the lung tissue and BALF, following administration of 100% oxygen and/or C5aRA. As shown in Figs. 4 and 7, hyperoxia significantly induced TNF-α, IL-6 and MCP-1 expression in lung tissue and BALF. The hyperoxia-induced TNF-α, IL-6 and MCP-1 expression in lung tissue and BALF were significantly suppressed following treatment with C5aRA. These data confirmed the other findings of the present study. C5a-C5aR action accelerated hyperoxia-induced lung injury and the hyperoxic lung injury was attenuated by treatment with C5aRA. However, as a novel therapeutic strategy for hyperoxic lung injury, the treatment with C5aRA still requires further investigation.

The present study has demonstrated for the first time, to the best of our knowledge, a crucial role of C5a-C5aR action in the acceleration of hyperoxia-induced lung injury and provides a rationale for the potential use of C5aRA in clinical practice to treat acute exacerbation of hyperoxic lung injury.
